# Arrangements with the NHS for providing healthcare services: do they improve financial performance of private for-profit hospitals in Spain?

**DOI:** 10.1186/s13561-021-00304-4

**Published:** 2021-03-10

**Authors:** María Victoria Ruiz-Mallorquí, Inmaculada Aguiar-Díaz, Beatriz González- López Valcárcel

**Affiliations:** 1 Department of Financial Economy and Accounting, Faculty of Economy, Business and Tourism, University of Las Palmas de Gran Canaria, 35017 Las Palmas, Spain; 2 Department of Quantitative Methods for Economics and Management, Faculty of Economy, Business and Tourism, University of Las Palmas de Gran Canaria, 35017 Las Palmas, Spain

**Keywords:** Hospitals, Financial performance, Private commissioning in healthcare

## Abstract

**Background:**

In developed countries around the world there is a trend to enhance the public-private collaboration in healthcare. In Spain, a decentralized country with a NHS funded with taxes and universal coverage, commissioning to for-profit private hospitals the production of healthcare services to specific patients that are publicly insured is a traditional practice. Around 43% of the for-profit private hospitals in Spain have a commissioning agreement with the NHS to diagnose or treat patients on public tariffs. These revenues represent 26% of the total revenues of private for-profit hospitals. The research question of this study is if commissioning with the NHS improves the financial performance of private-for-profit hospitals in Spain.

**Methods:**

With a long panel (2000–2017) of for-profit hospitals we estimate a model for the financial performance (return on assets) using commissioning as main explanatory variable and other variables as control (variables financial indicators and structural information). Specific models are estimated for subgroups of hospitals according to size and specialization. The models are estimated by panel regression with fixed effects and GMM as robustness.

**Results:**

Private for-profit hospitals that have commissioning with NHS obtain higher financial performance than no-commissioning hospitals. This effect varies depending on hospital size and type (hospital specialization), the advantage being more relevant for general hospitals and particularly for hospital with at least 50 beds.

**Conclusions:**

Commissioning with the NHS is a promising source of financial profitability for general acute private for-profit hospitals. The evidence provided by this study may orientate the NHS in the regulation and negotiation of commissioning contracts in healthcare.

**Supplementary Information:**

The online version contains supplementary material available at 10.1186/s13561-021-00304-4.

## Introduction

The public sector has adopted different management techniques from the private sector in the 1980’s, giving rise to what is now known as *New Public Management* [1, 2], which includes outsourcing and externalization of many public services. Outsourcing implies the separation of different activities, which belong to a production process of a public company and allows firms in the private sector to carry out all or part of them. Along this line, developed countries are delivering healthcare services in collaboration between public and private sectors [29, 38]. It can take on several forms, and one of them, called *commissioning,* is traditionally the most common practice in the Spanish healthcare system.

In Spain, there are different public financing schemes for privately produced health services. They comprise global financing agreements and specific agreements for delimited and defined groups of patients, with payment for activity based on individual services. The latter type, which we will call commissioning, is the one we analyse in this paper. Although there are other types of public-private collaboration in Spain, in this study we focus on traditional commissioning arrangements, consisting in contracting out with private hospitals so that the public supplier (autonomous government) can send patients for specified healthcare services performed at a regulated fixed tariff. This kind of traditional commissioning has been defined as “supplementary network covering some diagnostic tests and procedures, elective surgery in the context of waiting list reduction programmes, palliative care, long-term care and non-acute mental health care” [9]. We exclude from the definition of commissioning global funding agreements with a hospital and healthcare to civil servants covered by a private insurance company.

Private production represents 11.6% of public healthcare expenditures in Spain, including commissioning and other agreements. According to the National Catalog of Hospitals (CNH) around 43% of private for-profit hospitals were involved in public healthcare commissioning in 2018. Public funding (excluding the civil servants’ s vouchers) represented 26% of revenue for private-for-profit hospitals in 2016 [22].

The empirical evidence on commissioning in the healthcare arena has focused on the impact of hiring or contracting public agents (improved efficiency and greater quality, cost reduction). Nevertheless, very few references in the literature analyze commissioning with a view from the private hospital and its impact on the financial and economic situation of the hospital.

In this context, the aim of this study is to analyze if commissioning with the NHS improves the financial performance of private-for-profit hospitals in Spain by considering the possible moderating effect of hospital size and hospital type according to specialization in the commissioning-performance relationship. For-profit-private hospitals have not received much attention in the literature on healthcare economics, which suggest that the prevalence of for-profit ownership depends on the power of the market or the lower production costs for greater efficiency or improved access to capital [23].

Our empirical analysis uses panel data during the period 2000–2017 and is taken from financial-economic and healthcare sources. Spain offers an appealing opportunity for studies of this nature because of the presence of universal coverage within the NHS and public financing (and without copayments for hospital care). Even though the most of the healthcare services are provided by public centres with its own resources, there are nonetheless a significant number of commissioned hospitals. The main reason for this situation is that the Public Administration is unable to meet with its own resources the increasing demand for healthcare services resulting from universal healthcare for all of the public, which began in 1986. It should be noted that several important changes have occurred during the period of study: 1) Since 2002 all the 17 autonomous communities have full autonomy^1^ for deciding with who and how to commission other hospitals and at what tariffs; 2) The economic crisis which began in 2008 led to substantial budget cuts in public healthcare, and as a collateral effect, the rise of the private hospital sector; 3) The increasing concentration of private hospitals in the last decade as a strategy for growth and improvement of the negotiating position with insurance companies, which are their main clients.

Our results reveal that private-for-profit hospitals, which are commissioned by the NHS, show better financial performance than those who do not do it, and that hospital size and typology are a mediating factor. General hospitals with more than 50 beds benefit the most.

The rest of this paper is structured as follows. Section 2 offers a theoretical background about public-private collaboration and the impact of the commissioning on the financial performance of the private hospitals. The third section presents the data and reviews methodology. The next section offers a descriptive analysis and the results of the econometric study, while section 5 discusses the results, their limitations and the implications of the study. The paper ends with some conclusions.

## Theoretical arguments and hipotheses

Studies in the literature concerning public-private collaboration in the healthcare arena have used several different approaches [1, 10, 39], including: the theory of public choice, which justifies Public-Private Partnership (PPP) from a public sector perspective; the theory of ownership rights, which compares the efficiency of public centers with private ones; and the theory of transaction costs, which is the leading argument from the private sector point of view, in which this paper focuses.

The theory of transaction costs defines transaction costs as the comparative costs of planning, adapting and controlling the completion of tasks, under the structures of alternative government [42]. That is, transaction costs are essentially management costs associated with contracting a service. Factors which play a part in transaction costs are limited information and uncertainty [42], which can prevent contracts from being completely defined. At the same time, Williamson [42] points out two characteristics of services related with transaction costs: the level of specialization of the assets and the measurement of the service. Specialization level refers to the need for specific investments to deliver the service, while the second is concerned with the difficulty of measuring results and/or controlling the needed activities to deliver them [11, 32]. Greater profits would be given in technical services characterized by need for lower specialization and are therefore easier to measure, while the opposite occurs for more complex services at a higher level of specialization bringing lower profits and measurement difficulties [39]. Similarly, two types of transaction costs can be identified [28]: *Ex ante* and *Ex post.* The first one refers to search costs and negotiation, while the second type includes the costs of controlling the contractual fulfilment by each part, and the costs of fulfilling contracts in case of disputes of an incomplete contractual specification. Stable/fixed contracts could lower both *Ex ante* and *Ex post* transaction costs.

The theory of transaction costs has been applied to private firms, and to explain decisions made in public service [42]. Public agencies can develop internal production mechanisms, but external ones as well, and this last group requires contracts that reduce the risk associated with the uncertainty that is generated from the asymmetries of information between each sector. These contracts, amongst others, can be developed with private agents by means of privatization of specific services. In this way, the transaction cost perspective implies that the new competition from the private sector does not always lead to a reduction of costs, as it will depend on the obtained profits being greater or not to the costs associated with the definition and proper supervision of the contracts [41].

Another perspective to solve the asymmetries of information and the incomplete contracts is related to reputation. In this sense, Atanasov et al. [5] assert that “reputation helps enforce contracts, because existing or potential counterparties can respond to a breach of contract by terminating or adjusting the terms of their business relationship and causing the breaching party to lose its entire stream of quasi-rents”(2216). Klein and Leffer [25], in their seminal paper, indicate as economists consider reputation a private mechanism to assure contract performance. Regarding the public services, Doni [17] develop a theoretical model in where he shows the importance of reputation in the awarding procedures in order to assure the quality of the services and to avoid the distortion of firm incentives. Empirically, different works have demonstrated the role of reputation in contracts between public and private sector [6, 24, 37].

The high amount of uncertainty in the healthcare arena leads to incomplete contracts and requires successive renewals, which is an explanation for their high costs [27]. Given that hospitals are complex organizations, which offer both low and highly specific services, there is uncertainty about the advantages of privatization in practice [39].

On the other hand, commissioning agreements allow the private sector an opportunity to increase potential demand, which could generate higher revenue and contribute to improved hospital performance, as long as they are able to contain costs and align public tariffs for commissioned services. Consequently, if commissioned private hospitals are able to reduce costs, even though at the cost of lowered quality of service, the commissioning arrangement would also have a positive effect on its financial performance. Participation in a commissioning arrangement must create value for the for-profit private hospitals, otherwise they would not continue with the arrangement. Along this line, the assumption of rational behaviour in organizations implies that those hospitals, which enter into the commissioning arrangement will be more profitable than if they do not.

Hardly any references in the literature analyze how a commissioning arrangement for the delivery of healthcare services influences financial performance of a commissioned hospital. As previously mentioned, we expect that the effect of being a commissioned hospital is neutral (not significant) or positive for the ROA of a hospital. Nevertheless, it is plausible that the effect is heterogeneous according to hospital characteristics, specifically size and hospital specialization. Hospital size can play a moderating role on the relation between commissioning and financial performance. If the commissioning arrangement improves the expected profitability of large hospitals more than small ones is an empirical question, which we will explore in this study. Indeed, hospital size (beds and assets) approximates its negotiating stance [33].

It is also reasonable to assume that both economies of scale and the actual profitability of the commissioning arrangement are heterogeneous according to hospital specialization because specialization is associated to transaction costs. Greater profits are obtained in technical services characterized by lower speciality and greater ease of measure, while lower profits are obtained for more complex services that require a higher level of speciality and are more difficult to measure. Consequently, we would expect that general hospitals would get higher profitability from the commissioning arrangement than other hospitals.

## Material and methods

### Data

A combination of healthcare, economic-financial and demographic databases has been used to create a panel of hospitals for the period 2000–2017. We start from the National Catalog of Hospitals (CNH) [30], annual publication of the Ministry of Health that contains the detailed information of structure and technological endowment of all hospitals in the country, public and private, including the existence of a contract with the NHS. According to the CNH 2017 there are 913 hospitals, of which 317 (34.72%) are private for-profit. The initial sample consists of an unbalanced panel of 5681 observations corresponding to 449 hospitals. The number of hospitals per year ranges from 304 to 336, due to the inflows and outflows produced throughout the period.

The CNH base has been linked to the financial information of the hospitals, coming from the SABI database (Iberian Balance Analysis System). SABI contains the information of the commercial register on the annual accounts of the companies. All companies are required to deliver their accounts, and SABI collects them. Therefore, SABI includes only hospitals with legal personality. In order to ensure the reliability of the information and its tracking throughout the entire period, the Tax Identification Number of each hospital has been previously located. In this way, cases of change of corporate name are detected, among other causes due to their incorporation into a hospital group. Once the companies are located in the SABI database and the existence of non-consolidated financial information has been confirmed, hospitals for which the necessary information is not available have been removed from the sample. Likewise, two forms of public-private collaboration, hospitals in the Catalan public utilization network and administrative concessions under the “Alzira model” have been eliminated.^2^ This has reduced the final sample to an unbalanced panel of 3284 observations corresponding to 286 hospitals.

In addition, demographic data of the National Institute of Statistics (INE) have been used to obtain variables concerning the hospital environment (see below).

### Variables

#### Dependent variable: financial performance

Given the nature of the hospitals included in this study, it is assumed that they have as a financial objective the profitability. As in previous studies, in this work the financial performance of hospitals has been approximated through ROA or economic profitability (e g, [8, 12, 15, 20, 36, 40, 43]). Specifically, the ROA has been calculated by ratio between the result before interest and taxes, known in the financial literature as EBIT (Earnings Before Interest and Taxes), and total assets.

### Explanatory variables

The explanatory variable of interest is the dummy (commissioning) of the existence of a contract signed with the government of the autonomous community for the provision of specific health services to publicly insured patients, which are transferred to the private center.

To approximate the size, the logarithm of the number of hospital beds installed has been used. As a robustness, the subsample of hospitals with at least 50 beds has been considered. Finally, the type of hospital has been considered in two subsamples: general and medium and long stay. The former includes maternal and pediatric and the latter integrates geriatric and psychiatric.

### Control variables

First, a standardized index of technological endowment of the hospital has been created for general and surgical hospitals, with CNH data on the endowments of units of 10 types of high-tech diagnostic and therapeutic equipment. This index has been calculated using a principal component analysis of the number of available devices.^3^ For surgical hospitals, only the different types of devices have been considered in binary form (having or not having), excluding those very rare in Spanish surgical hospitals (PET, CT-PET and linear accelerators). The first principal component of each of the analyzes has been standardized so that the average hospital has a zero score every year. For non-general or surgical hospitals, we have set the technological index equal to zero. This technological index has been introduced in quadratic form in the models in order to explore a possible non-linear relationship.

Second, according to previous studies on hospital financial performance, the age, the level of indebtedness, the economic structure and the fixed effect of the year have been considered as control variables. The age is used in the financial literature as a proxy of experience. As companies gain more experience, they are able to add methods that improve their productivity. Organizational learning makes it possible to identify recurring problem-solving procedures, reducing the time and effort used [14]. The age of the hospital is computed as the number of years since its establishment.^4^ The leverage is an indicator of the capital structure and represents the company’s ability to make use of external financing sources to finance its investments, so it can condition the possibility of undertaking new projects. To measure the leverage, the total volume of debt has been relativized by the total asset [4, 20], which allows comparing companies of different sizes. The economic structure has been approximated by the tangibility obtained by quotient between the book value of the investments that make up the tangible assets and the total assets [34]. The relative volume of tangible assets is a benchmark indicator for obtaining financial resources to the extent that it represents a potential source of collateral for the debt. In the case of hospitals, which require significant investments in infrastructure, both general and clinical, high tangibility has advantages and disadvantages, since high volumes of fixed assets represent greater borrowing capacity, but also higher fixed costs. Finally, time fixed effects (year), annual dummies for the years 2001 to 2017 have been included (e.g., [43]).

Lastly, it should be noted that some studies have considered control variables related to location [7, 16, 20, 21, 31, 35], as well as the type of city in which the hospital is located [18, 21]. However, it is not possible to include them in the models estimated by fixed effects as they are time invariant for each hospital. However, two proxies about the location has been incorporated to approximate the potential demand for medical services in the hospital’s area of influence and to instrument the commissioning, once we consider the possibility that it is endogenous (see below, section 3.3).^5^

### Models and identification strategy

We have estimated the following panel model with fixed effects of hospital and year:

$$ {y}_{it}=\alpha +{Z}_{it}\delta +{X}_{it}\beta +{\eta}_i+{\zeta}_t+{\varepsilon}_{it} $$

where the dependent variable *y*_*it*_ is the ROA previously defined for hospital i in year t. Z includes the explanatory variables of interest, and specifically the existence of a commissioning contract. Although the agreement is quite stable over time (year-to-year persistence of commissioning with the same hospitals), there is sufficient within-hospital variability, given the temporal length of the panel (see Table 3), to estimate with fixed effects; X includes the covariates used as controls, *ε*_*it*_ are the random errors iid Normal with mean 0 and variance ${\sigma}_{\varepsilon}^2$, *η*_*i*_ are the unorbserved hospital effects that are assumed to be realizations of an i.i.d. process with mean 0 and variance ${\sigma}_{\eta}^2$, and *ζ*_*t*_ are time fixed effects. *η*_*i*_ and *ζ*_*t*_ are assumed to be independent of *ε*_*it*_. On the other hand, the existence of extreme values in some of the variables might overweight the influence of possible spurious outliers on the results. To avoid it, and following the financial literature (e.g., [43]), we proceeded to winsorize the ROA at the top and bottom 1% and the leverage at the top 1% of their respective distribution. Stata 15 was used for the estimation.

To analyze the possible mediating effect of hospital size and type on the effect of commissioning on financial performance, the model has been estimated for specific subsamples by purpose and size. Specifically, distinguishing general hospitals from the rest, and only for hospitals of minimum size 50 beds.

The advantage of the fixed effects model is that by canceling the unobservable individual heterogeneity of the hospital (and of the year) by making the demeaning (that is, when calculating the variations of each year with respect to the time average of the variable for that hospital), the estimate of the commissioning effect would be consistent even if commissioning was not exogenous, but associated to intrinsic characteristics of the hospital. However, it could happen that commissioning is endogenous in the sense of presenting stochastic dependence with the time varying error. This would be so if some unobservable determinants of the ROA that vary temporarily and are part of the error are related to the agreement. Hospitals must meet certain requirements in order to be eligible for a commissioning agreement in Spain Some of these requirements, which vary among Autonomous Communities, are financial (volume of sales, net income, net equity), but profitability is not one of them.

To consider this possibility, and as proof of robustness, the model has been estimated by the Generalized Method of Moments (GMM) [3], which adds the lagged ROA to the equation. We used instrumental variables based on demographic information of the municipality in which the hospital is located and the supply of public beds in it. Specifically, two instruments are considered. First, the number of public beds in the municipality where the hospital is located. Second, a dummy = 1 for municipalities under 50,000 inhabitants without public hospital of the same type (i.e. general, surgical, medium and long stay, psychiatric, other monographic).

## Results

### Descriptive analysis

According to the CNH, there were 305 for-profit hospitals in Spain in 2000, 336 in 2009 and 324 in 2017. The private sector represents between 41 and 44% of the total number of functioning hospitals during the period 2000–2017, although the number of beds is much less, not even reaching 20%. In 2017 the number of beds in the private- for-profit sector was 29,874 out of a total of 158,269, approximately 18.87%. These data clearly show that average bed size in public hospitals is double the number in private ones. Table 1 presents the distribution of the population (CNH) and the sample (complete SABI economic-financial information) by years and commissioning status. As can be observed, the final sample represents approximately 58% of the population, with commissioned hospitals throughout the period being 45%.

**Table 1:** Sample distribution by years

Year	Population (CNH)	Final sample (SABI)	Number Commission	% Commission	Year	Population (CNH)	Final sample (SABI)	Number Commission	% Commission
2000	305	175	89	50.85	2009	336	197	88	44.67
2001	308	182	87	47.80	2010	330	196	87	44,38
2002	304	189	89	47.08	2011	315	190	87	45.78
2003	304	188	89	47.34	2012	312	183	83	45.35
2004	312	185	88	47.56	2013	313	179	83	46.36
2005	313	190	76	40.00	2014	313	181	82	45.30
2006	318	193	80	41.45	2015	312	173	78	45.08
2007	324	183	78	42.62	2016	312	160	75	46.87
2008	326	196	85	43.36	2017	324	144	67	46.52
**N.obs**						**5.681**	**3.284**	**1.491**	**45.40**

Source: Own elaboration based on CNH and SABI

There are 286 different hospitals that have been included for at least one year in the final SABI sample database. There are centres that were eliminated because of missing values. As regards hospital size, Table 2 divides hospitals into three groups: small (less than 50 beds), medium (between 50 and 100 beds) and large (more than 100 beds).

**Table 2:** Sample distribution by type and size

	Total sample		Commissioning	
**Type**	N Obs.	%	N Obs.	**% (over type)**
General	2091	63.67	1.055	50.45
Surgical	298	9.07	142	47.65
Psychiatric(a)	341	10.38	94	27.56
Geriatric & long stay(a)	410	12.48	182	44.39
Other monographic	144	4.38	18	12.5
Size	N Obs.	%	N Obs.	**% (over size)**
Small (Less 50 beds)	1101	33.53	335	30.42
Medium (Between 50 and 100)	1131	34.44	527	46.59
Big (More than 100 beds)	1052	32.03	629	59.79
**Total**	**3284**	**100**	**1491**	**45.40**

Obs.: observations refer to hospital-year

(a) Psychiatric and Geriatric & long stay are included in the category “Medium and Long stay” in the models

Source: Own elaboration

The distribution of observations (hospital-year) is nearly identical for the three groups, although the highest percentage of commissioned hospitals is found in the largest category, followed by the medium group and finally the small hospital group. General hospitals (including maternity-paediatrics) accumulate 64% of the panel observations, as opposed to surgical, at 9%, while psychiatric and geriatric together make up 22%. The percentage of commissioning arrangements in each type of hospital greater than 44%, except for psychiatric (27%), and for other specialities (12,5%).

Of the 286, 125 (47.53%) were never classified as commissioned, 81 (30.80%) had always been for the observation period and the remaining 57 (21.67%) were commissioned for some years during the sampling period. Classification by typology and commissioning history is considered in Table 3. As regards hospital types, the “other specialties” reveal limited presence in commissioned hospitals, and the commissioning agreements are more stable than the other types, as there is just one hospital in this category that has changed commissioning type during the observation period (and in the case of surgical centres, three hospitals (Table 3). This fact led us to estimate specific models only for general hospitals and for the group composed by psychiatric, geriatric and long stay hospitals (from now on we will call them Medium & long stay hospitals).

**Table 3:** Commissioning history during the period, by hospital type^a^

Type	Never commissioning		Always commissioning		Commissioning in some years		Total
	N	%	N	%	N	%	N
General	65	42.48	51	33.33	37	24.19	153
Surgical	14	43.75	15	46.87	3	9.38	32
Psychiatric(a)	15	60.00	2	8.00	8	32.00	25
Geriatric & long stay(a)	18	48.65	11	29.73	8	21.62	37
Other monographic	13	81.25	2	12.50	1	6.25	16
Total	125	47.53	81	30.80	57	21.67	263

^a^There are only 263 hospitals included in Table 3 because the remaining 23 changed their classification at least once during the study period. The most common changes occurred in geriatrics, long term stay and psychiatric, and those between surgical and general

The % are calculated over the total of their type

(a) Psychiatric and Geriatric & long stay are included in the category “Medium and Long stay” in the models

Source: Own elaboration

There are significant differences in the prevalence of commissioned private for-profit hospitals by autonomous communities (Additional file 1), with figures ranging from 18.7% in Andalucía, 74% in Galicia, and 100% in La Rioja.

Tables 4 and 5 show the descriptive statistics and the correlation matrix of the continuous variables, respectively. The average number of beds per hospital in the sample is 96.

**Table 4:** Descriptive statistics

	Average	S.D.	Median	Mín	Max	Average Comm.	Average No comm.	Test for average differences
ROA (%)	4.57	13.65	5.02	−60.52	40.18	5.07	4.15	− 1.93^**^
Size (Log beds)	4.21	0.87	4.30	1.38	6.83	4.46	4.00	−15.40^***^
Age (Log years)	2.97	0.83	3.09	0	4.76	3.04	2.91	−4.60^***^
Technological index	0.00	0.77	0	−1.104	3.08	0.12	−0.09	−8.18^***^
Leverage (%)	58.68	34.30	55.12	0	216.2	60.43	57.24	−2.65^***^
Tangibility (%)	42.21	24.87	41.57	0	100.0	43.70	40.97	−3.13^***^
Bed number	95.79	91.72	74	4	931.0	113	82	−9.57^***^

**, ***: significant to 5 and 1%, respectively

Comm = commissioning hospitals

Source: Own elaboration

**Table 5:** Correlation matrix between continuous variables

	1	2	3	4	5	6
1. ROA	1					
2. Size (Log beds)	0.0888^***^	1				
3. Age (Log years)	0.0616^***^	0.2013^***^	1			
4. Technological index	0.0793^***^	0.5094 ^***^	0.0867^***^	1		
5. Leverage	−0.4077^***^	−0.0798^***^	−0.2379^***^	− 0.0719^***^	1	
6. Tangilbility	−0.0717^***^	−0.0286	0.1461^***^	−0.0056	− 0.0409^**^	1

**, ***: significant to 5 and 1%, respectively

Source: Own elaboration

The technological index has zero average (as it is standardized); its variance is less than one because all of the non-general nor surgical centres have an index = 0 (consequently, their intragroup variance is zero). Leverage (indebtedness level) is at 70% of assets in average and the tangibility (tangible fixed assets as a proportion of total assets) is 42%.

The correlation displays low values for the explanatory variables and consequently there are no difficulties with respect to multicolinearity.

Table 6 presents mean ROA and its standard deviation for the different subgroups of hospitals by size and type, and the “student’s t test for equal means between commissioned and non-commissioned groups.

**Table 6:** ROA for Commissioning hospitals versus no commissioning (Average-S.D) %

Sample	Total	Commissioning	No commissioning	Two-tail t-test
All	4.57–13.65	5.07–12.99	4.15–14.16	− 1.93^*^
Small(^a^)	2.44–15.72	3.19–16.15	2.12–15.52	− 1.04
Medium (^a^)	5.46–13.77	6.32–14.09	4.75–13.47	−1.85^*^
Big(^a^)	5.76–10.80	4.96–9.85	6.96–12.00	2.96^***^
General	3.58–13.17	4.89–12.62	2.25–13.56	−4.59^***^
Surgical	0.63–14.36	1.84–13.89	2.47–14.80	0.37
Psychiatric(a)	8.30–11.99	6.83–11.99	8.86–13.01	1.31
Geriatric & long stay(a)	8.43–15.12	7.50–14.53	9.17–15.57	1.11
Other monographic	4.13–12.92	8.16–9.81	3.55–13.23	−1.42

*, ***: significant to 10 and 1%, respectively

^a^Small: < 50 beds; Medium: between 50 and 100 beds; Big: > = 50 beds

(a) Psychiatric and Geriatric & long stay are included in the category “Medium and Long stay” in the models

Source: Own elaboration

Profitability of commissioned hospitals is greater than non-commissioned ones for small and medium-sized hospitals, although the opposite occurs for hospitals with more than 100 beds (a figure which is close to the sample mean). These figures suggest that the relationship between commissioned hospitals and profitability depends on hospital size.

The relationship between profitability and size according to the commissioned status can be reflected by the differences in hospital size by hospital. In general, in acute care hospitals, as well as in other specialities, mean profitability of the commissioned hospitals is greater than the non-commissioned counterparts, while in long term care (psychiatric and geriatric) the difference is not significant, even though average profitability is greater for the non-commissioned hospitals. Geriatric hospitals and psychiatric ones are more profitable than the rest. However, in commissioned hospitals, the other monographic hospitals have the highest profitability.

### Estimation results

Table 7 presents the results for the within-group estimators. M1 and M2 are for all hospitals, M1 reflects the entire sample and M2 are for the subsample of hospitals with more than 50 beds. M3-M4 and M5-M6 present the estimated models corresponding to general hospitals and to mid and long term care (geriatric, psychiatric), respectively.

**Table 7:** Estimated models for the whole sample and subsamples

	M1	M2	M3	M4	M5	M6
Sample	All hospitals	All hospitals > = 50 beds	General All	General > = 50 beds	Medium & long stay	Medium & long stay > = 50 beds
Commissioning	0.0332^***^	0.0317 ^***^	0.0223^**^	0.0335^***^	0.0400^**^	0.0209
	(0.0083)	(0.0089)	(0.0100)	(0.0110)	(0.0181)	(0.0189)
Beds (log)	0.0328^*^	0.0341^*^	0.0519	0.0500^**^	0.0722^***^	0.0852
	(0.0100) ^**^	(0.0203)	(0.0162)^**^	(0.0231)	(0.0199)	(− 0.0546)
Age (log)	0.0108	0.0083	0.0187^*^	0.0032	0.0245	0.0756 ^***^
	(0.0080)	(− 0.0091)	(0.0097)	(0.0105)	−0.0193	(0.0216)
Techn.index	0.0172^*^	0.0203^**^	0.0225^**^	0.0260^**^	–	–
	(0.0092)	(0.0101)	(0.0110)	(0.0116)		
Techn.index^2^	−0.0125 ^***^	− 0.0143^***^	− 0.0157^***^	− 0.0174^***^	–	–
	(0.0044)	(0.0045)	(0.0049)	(0.0051)		
Leverage	−0.2264^***^	−0.2196^***^	− 0.2276^***^	−0.2213^***^	− 0.2570^***^	−0.2264^***^
	(0.0090)	(0.0108)	(0.0110)	(0.0134)	(0.0201)	(0.0217)
Tangibility	−0.0368^***^	−0.0641 ^***^	− 0.0800^***^	−0.0863^***^	− 0.0012	−0.0273
	(0.0133)	(0.0155)	(0.0172)	(0.0197)	(−0.0275)	(−0.0302)
Constant	0.0249	0.0193	−0.0715	−0.041	− 0.1455	−0.3807
	(0.0489)	(0.1006)	(0.0754)	(0.1137)	(0.1069)	(0.2685)
Years^a^	Yes	Yes	Yes	Yes	Yes	Yes
N Observations	3284	2183	2091	1467	751	532
R^2^ within	0.2076	0.2112	0.2235	0.2170	0.2541	0.2527
R^2^ between	0.2326	0.1092	0.2277	0.1155	0.0922	0.0217
R^2^ overall	0.1732	0.1397	0.1631	0.1118	0.1515	0.1121
rho	0.5273	0.5154	0.5673	0.5531	0.5407	0.6770

*, **, ***: significant to 10, 5 and 1%, respectively . Standard error in parentheses

^a^Years estimation results can see in Additional file 2

Source: Own elaboration

Dependent variable: ROA. Estimation method: panel regression with fixed effects

The commissioning variable is significant and positive in all models except in Model 6 (Medium & long stay whit at least 50 beds). Specifically, commissioning is highly significant (*p* < 0.01) in Models M1, M2 and M4 (full sample, hospitals with more than 50 beds and general hospitals larger than 50 beds). In M3 and M5 commissioning is also significant at 5% (*p*-value< 0.028). Regarding the size of the effect, coefficients range between 0.02 and 0.04. Commissioning increases the ROA between 2 and 4% for private hospitals.

Hospital size is significant and positive in all of the models except for M6. On the contrary, hospital age is only significant in this model. Technological index is significant and shows a quadratic effect. Its linear coefficient is positive (significant at 5% or 10%), while the quadratic coefficient is negative and significant at 1%.

Both leverage and tangibility are negative and highly significant in all of the models, with the exception of tangibility for those hospitals offering mid-term and long term care. Finally, fixed time effects (see Additional file 2 for detailed results), are negative and significant in most of the models starting from 2010, and especially for geriatric hospitals of mid-term care and psychiatric ones, which can be interpreted as a secondary effect of the economic crisis which has reduced the profitability of private hospitals.

The fixed time effects reveal that profitability have worsened starting in 2010, specifically for psychiatric hospitals in the years 2013–2015 (Additional file 2).

The results of the estimation by GMM (Table 8) regarding the effect of commissioning confirm the results of Table 7 only for the full sample and for general hospitals (M7, M9 and M10). For medium and long-stay hospitals, commissioning is not significant (M11 and M12) and negative. The effect and significance of the financial control variables is similar to that in the within-groups estimation. In regard to the remaining control variables, estimates are not so robust. Technological index has no significant effect while hospital age is significant and positive in three of the six models.

**Table 8:** GMM Estimated models for the whole sample and subsamples

	M7	M8	M9	M10	M11	M12
Sample	All hospitals	All hospitals > = 50 beds	General All	General > = 50 beds	Medium & large stay	Medium & large stay > = 50 beds
ROA (Lag1)	0.3157^***^	0.3431^***^	0.3673^***^	0.4325^***^	0.1586^***^	0.0938^**^
	(0.0238)	(0.0267)	(0.0295)	(0.0303)	(0.0426)	(0.0470)
Commissioning	0.0367^***^	0.0213	0.0458^***^	0.0281^*^	−0.0172	− 0.0178
	(0.0148)	(0.0155)	(0.0157)	(0.0153)	(0.0355)	(0.0370)
Beds (log)	0.0075	− 0.003	0.0150	0.0100	0.0469	−0.0059
	(− 0.0257)	(− 0.0216)	(− 0.0310)	(− 0.0299)	(− 0.0577)	0.0057
Age (log)	0.0213)^**^	0.0329^*^	0.0327	0.0137	−0.025	0.1038^**^
	(0.0181	(0.0193)	(0.0203)	(0.0180)	(0.0393)	(0.0489)
Technol.index	0.0049	0.0085	0.0047	0.0078	–	–
	(0.0151)	(0.0158)	(0.0149)	(0.0146)		
Technolog.index^2^	−0.005	− 0.009	− 0.005	− 0.010	–	–
	(0.0071)	(0.0071)	(0.007)	(0.0066)		
Leverage	−0.276^***^	− 0.236^***^	− 0.253^***^	− 0.168^***^	−0.260^***^	− 0.257^***^
	(0.0149)	(0.0168)	(0.0171)	(0.0191)	(0.0251)	(0.0263)
Tangibility	−0.068^***^	−0.105^***^	− 0.094^***^	−0.075^***^	− 0.045	−0.058
	(0.0219)	(0.0243)	(0.0262)	(0.0282)	(0.0384)	(0.0417)
Constant	0.0659	0.0811	0.0048	0.0292	0.1385	−0.0169
	(0.1016)	(0.1378)	(0.1166)	(0.1579)	(0.1901)	(0.3071)
Years^a^	Yes	Yes	Yes	Yes	Yes	Yes
N Observations	2582	1767	1668	1201	589	430
Wald Chi2	755.77	627.00	539.32	520.34	244.89	165.97

*, **, ***: significant to 10, 5 and 1%, respectively . Standard error in parentheses

^a^Years estimation results can see in Additional file 3

Source: Own elaboration

Dependent variable: ROA

## Discussion

Univariate description of the sample shows that profitability for mid and long stay hospitals (including geriatric and psychiatric hospitals) in Spain is much higher than for general and surgical ones. On the other hand, variability of ROA among hospitals in the study period is very high, as is the within-hospital variability of the ROA.

Based on our modelling results, we can assert that a commissioning arrangement helps improve profitability of private for-profit hospitals, although this relationship is influenced by size and the type of healthcare provided by the hospital. Thus, the ROA could be around 2–4% higher for commissioned hospitals. This result is robust for general hospitals, as fixed effects within-group estimates and GMM estimates agree. On the contrary, for mid and long stay hospitals, the results of the fixed-effect models are not confirmed by the GMM estimation. The effect of commissioning on ROA is smaller for larger general hospitals than for the full sample of general hospitals.

Control financial variables have the expected signs and are robust.

Models M1-M6 show that profitability of for-profit Spanish hospitals worsened during the economic crisis, from 2010. Nevertheless, a change of effect during these years of economic crisis due to the commissioning agreement (interaction term) was not found significant (results not shown).

Commissioning arrangements in Spain are stable, and many hospitals have entered this collaboration during the 18-year study period, while only 57 have joined to leave later during the study period. Stability reduces ex ante transaction costs (there is an initial negotiation and then tariffs are essentially adjusted by a price index). Ex post control costs are more limited for commissioned standardized procedures, as diagnostic tests, surgical interventions for elective surgery, or physical therapy sessions. In addition, as it is the public provider who refers patients specifically to the private commissioned center, the “capture of the regulator” is avoided. However, in practice the compatibility of the work of doctors in both networks, public and private, could cause deviations and generate commissioned activity not indicated clinically. This in turn might improve the financial performance of commissioned centers without providing therapeutic value, or only very marginally. A common practice in recent years is to hire facilities (operating rooms) and auxiliary staff of the private center but perform surgical interventions with surgeons of the public network under salary. In this case, the hypothetical conflict of interest problems would disappear.

In the USA, panel data models showed that occupation rate, size, availability of technology and market power were significant predictors of hospital profitability [33]. The database in our study does not contain market power measures and the CNH does not provide clinical information. We have calculated a Herfindahl-Hirschman Index (HHI) concentration index based on the number of beds at provincial level and we have used it as an additional control variable, but it is not significant in Spain. Similar to the Rosko et al. study, the economic crisis in Spain also affected hospital profitability (fixed time effects from the years of the crisis are negative and significant). While a reference to the Affordable Care Act under Obama in the United States which extended the Medicaid program is not completely comparable, it did have a positive impact on hospital profitability in those states where growth in hospitals did occur, suggesting that in some way treating new patients that are financed by public programs also improved expected profitability in the United States, as in Spain.

A review of commissioning in the case of England after the passing of the Health and Social Care Act 2012 reveals that it has been seen as a type of *marketisation,* which does not necessarily lead to privatization [26], as the attempts to maximize the value for money in times of austerity can lower expected profit margins by reducing prices. As in Spain, prices are fixed in a top-down manner and are based on activity. One of the remaining challenges in Spain is to fix tariffs based more on its own proper cost accounting of production and less on copying tariffs from other territories with adjustments for inflation. Other forms of financing based on results are yet to be considered.

The present work is novel on a topic which has been surprisingly ignored in the literature. A strong point of the study is the length of the panel data, which covers a time period from 2000 to 2017, allowing changes in size, technological structure, and financial variables of private for-profit hospitals to be seen. In addition, there have been changes in commissioning arrangements, signing of new contracts or finalizing current ones during the study. Another strength of the study is that the database used comes from the population register (National Catalogue of Hospitals).

Despite these advantages there are limitations in the study. The following two paragraphs address these limitations.

As for the group of hospitals analyzed, although it is based on a population register (CNH), the effective criterion of inclusion in the sample is that the hospitals present the accounts in the commercial register, which excludes hospitals belonging to groups, when consolidated accounts are presented for the entire holding. Therefore, the study is limited to independent hospitals, which limits external validity for the entire NHS. It is not a serious limitation as it might seem because large groups of hospitals generally do not contract with the public system. The disappearance of individual hospitals from the database may be the effect not of their disappearance but of their absorption or purchase by a hospital group. In this sense, one might think that the model has attrition bias (hospitals do not disappear at random, but perhaps attrition could be endogenous, related to non-measurable characteristics that affect profitability and vary temporarily).

Unfortunately, there is no information either on the type of outputs or inputs that are purchased at hospital level. Also, there is no available information on the fees, tariffs or payment strategy by the Autonomous Communities. There are variables explaining the profitability (and in particular, the differential profitability of the commissioning hospitals) that it has not been possible to consider due to lack of information, such as the length of the public waiting list (which will influence the level of commissioned activity more than the fact of opening new agreements), the productivity of the public sector, the specific morbidity associated with the services that are contracted and some of the organizational, management and structure variables that usually enter the models that explain the financial performance with longitudinal data from hospitals in the USA. Those variables might contribute to improve the explanatory capacity of the estimated models.

Despite the aforementioned limitations, we have got robust estimates of the main effects we were analyzing regarding to the effect of commissioning on profitability.

## Conclusion

In conclusion when a general hospital (acute care), and particularly if it is above a 50 beds threshold, decides to be commissioned by the NHS, it would expect an improvement in its financial performance. There is no conclusive evidence that it will improve expected financial performance for hospitals that provide mid and long-term care.

## Supplementary Information

**Additional file 1.**

**Additional file 2.**

**Additional file 3.**

## Data Availability

All data supporting the results and conclusions in this study are available on request to the authors.

## Abbreviations

CNH: National Catalog of Hospitals
EBIT: Earnings before interest and taxes
GMM: Generalized method of moments
NHS: National Health System
PPP: Public-Private Partnership
ROA: Return on assets

## Footnotes

1 Like other countries, Spain, “have opted to decentralize the responsibility for health care to lower levels of government and administrations” [19].

2 In Catalonia there is a hospital network for public use formed by the centers belonging to the public network and hospitals outside it. The “Alzira model” is a type of PPP consisting in an administrative concession regime, the private hospital provides full healthcare services to all the population publicly insured in a health area [13].

3 These devices are CT, Magnetic Resonance, Gamma camera, Hemodynamic Rooms, Digital Subtraction Angiology, linear accelerators, PET, CT-PET, mammography and bone densitometry.

4 Gapenski et al. [20] considered the age but they compute it from the accumulated depreciation of the plant.

5 Other studies [8, 15, 20, 43] have considered as control variables the attractiveness of the market, from the side of demand (income per capita), and/or of supply (e.g., degree of market concentration). The per capita income and the Herfindal-Hirdchman concentration index, both calculated at the provincial level, have been prepared and included in the models. However, these variables have not been significant so they do not contribute to improving the explanatory power of the model (results not reported).
