# Supplementary material for: Arrangements with the NHS for providing healthcare services: do they improve financial performance of private for-profit hospitals in Spain?
Source: Health Econ Rev. 2021 Mar 10;11:9. doi: 10.1186/s13561-021-00304-4 (PMC7944633; doi:10.1186/s13561-021-00304-4)
Supplement: Supplementary file 1 — Additional file 1. [file 13561_2021_304_MOESM1_ESM.docx]

| **Table A1. Sample distribution by Autonomous Community (AC)** | | | | | | |
| --- | --- | --- | --- | --- | --- | --- |
|  | **Autonomous Community** | **Private for-profit hospitals** | **%** | **Commissioning hospitals** | **% over AC** | |
| 1 | Andalusia | 545 | 16.60 | 102 | 18.72 | |
| 2 | Aragón | 59 | 1.80 | 45 | 76.27 | |
| 3 | Asturias | 102 | 3.11 | 32 | 31.37 | |
| 4 | Baleares | 140 | 4.26 | 69 | 49.29 | |
| 5 | Canarias | 202 | 6.15 | 134 | 66.34 | |
| 6 | Cantabria | 18 | 0.55 | 15 | 83.33 | |
| 7 | Castilla y León | 96 | 2.92 | 74 | 77.08 | |
| 8 | Castilla-La Mancha | 144 | 4.38 | 95 | 65.97 | |
| 9 | Catalonia | 628 | 19.12 | 85 | 13.54 | |
| 10 | Comunidad Valenciana | 239 | 7.28 | 97 | 40.59 | |
| 11 | Extremadura | 56 | 1.71 | 19 | 33.93 | |
| 12 | Galicia | 332 | 10.11 | 246 | 74.10 | |
| 13 | La Rioja | 22 | 0.67 | 22 | 100.00 | |
| 14 | Madrid | 319 | 9.71 | 202 | 63.32 | |
| 15 | Murcia | 144 | 4.38 | 120 | 83.33 | |
| 16 | Navarre | 35 | 1.07 | 13 | 37.14 | |
| 17 | Basque Country | 203 | 6.18 | 121 | 59.61 | |
|  | **Total** | **3,284** | **100** | **1,491** |  | |
|  | | | | | |  |
