# Supplementary material for: Arrangements with the NHS for providing healthcare services: do they improve financial performance of private for-profit hospitals in Spain?
Source: Health Econ Rev. 2021 Mar 10;11:9. doi: 10.1186/s13561-021-00304-4 (PMC7944633; doi:10.1186/s13561-021-00304-4)
Supplement: Supplementary file 2 — Additional file 2. [file 13561_2021_304_MOESM2_ESM.docx]

| **Table A2. Years estimation results in models M1-M6 (Continuation of Table 7)**  Dependent variable: ROA. Estimation method: panel regression fixed effects | | | | | | |
| --- | --- | --- | --- | --- | --- | --- |
|  | **M1** | **M2** | **M3** | **M4** | **M5** | **M6** |
| Sample | All hospitals | All hospitals  >=50 beds | General  All | General  >=50 beds | Medium & long stay | Medium & long stay  >=50 beds |
| Year 2001 | 0.0052 | 0.0092 | 0.0075 | 0.0131 | 0.0129 | 0.0264 |
|  | (0.0105) | (0.012) | (0.0123) | (0.0139) | (0.0285) | (0.0321) |
| Year 2002 | 0.0026 | 0.0011 | 0.0168 | 0.0127 | -0.0186 | -0.0073 |
|  | (0.0105) | (0.012) | (0.0123) | (0.014) | (0.0281) | (0.0317) |
| Year 2003 | 0.0199^*^ | 0.0232* | 0.0211^*^ | 0.0262^*^ | 0.0394 | 0.0399 |
|  | (0.0106) | (0.0121) | (0.0124) | (0.0140) | (0.0281) | (0.0316) |
| Year 2004 | 0.0049 | 0.0095 | 0.0073 | 0.0119 | 0.0122 | 0.0114 |
|  | (0.0108) | (0.0123) | (0.0127) | (0.0143) | (0.0283) | (0.0318) |
| Year 2005 | 0.0116 | 0.0141 | 0.0067 | 0.0159 | 0.0422 | 0.0310 |
|  | (0.0109) | (0.0124) | (0.0127) | (0.0142) | (0.0291) | (0.0335) |
| Year 2006 | 0.0076 | 0.0072 | 0.0089 | 0.0131 | 0.0237 | 0.0074 |
|  | (0.0109) | (0.0124) | (0.0128) | (0.0142) | (0.0292) | (0.0335) |
| Year 2007 | 0.0079 | -0.0022 | 0.0011 | -0.0036 | 0.0279 | 0.0126 |
|  | (0.0112) | (0.0127) | (0.0131) | (0.0145) | (0.0305) | (0.0347) |
| Year 2008 | 0.0123 | -0.000 | 0.0105 | 0.0080 | 0.0204 | 0.0002 |
|  | (0.0112) | (0.0125) | (0.0129) | (0.0142) | (0.0301) | (0.0347) |
| Year 2009 | -0.0014 | 0.0040 | 0.0154 | 0.0199 | -0.0162 | -0.0122 |
|  | (0.0114) | (0.0127) | (0.0133) | (0.0145) | (0.0307) | (0.0347) |
| Year 2010 | -0.0201^*^ | -0.0207^**^ | -0.0013^***^ | 0.0051^***^ | -0.0583^**^ | -0.0744^*^ |
|  | (0.0116) | (0.0128) | (0.0135) | (0.0147) | (0.0315) | (0.0352) |
| Year 2011 | -0.0208^*^ | -0.0265^**^ | -0.0107^**^ | -0.0083^***^ | -0.0479^**^ | -0.0645^**^ |
|  | (0.0119) | (0.0131) | (0.0140) | (0.0151) | (0.0320) | (0.0352) |
| Year 2012 | -0.0267^**^ | -0.0238^*^ | -0.0140^**^ | -0.0096^***^ | -0.0333^**^ | -0.0408^*^ |
|  | (0.0121) | (0.0134) | (0.0141) | (0.0153) | (0.0332) | (0.0361) |
| Year 2013 | -0.0348^***^ | -0.0248^**^ | -0.0238^**^ | -0.0085^**^ | -0.0809^*^ | -0.0861^*^ |
|  | (0.0123) | (0.0135) | (0.0143) | (0.0155) | (0.0336) | (0.0362) |
| Year 2014 | -0.0357^***^ | -0.0290^**^ | -0.0168 | -0.0135 | -0.0759 | -0.0751^**^ |
|  | (0.0125) | (0.0138) | (0.0145) | (0.0159) | (0.0338)^**^ | (0.0364) |
| Year 2015 | -0.0297^**^ | -0.0257^*^ | -0.0109^**^ | -0.0080^***^ | -0.0757 | -0.0953^*^ |
|  | (0.0128) | (0.0141) | (0.0150) | (0.0163) | (0.0344)^*^ | (0.0372) |
| Year 2016 | -0.0304^**^ | -0.0233 | -0.0124 | -0.0087 | -0.0621 | -0.0785^**^ |
|  | (0.0132) | (0.0147) | (0.0155) | (0.0171) | (0.0350)^*^ | (0.0378) |
| Year 2017 | -0.0466^***^ | -0.0316^**^ | -0.0369^**^ | -0.0142 | -0.0660 | -0.0887^**^ |
|  | (0.0138) | (0.0155) | (0.0163) | (0.0182) | (0.0356)^*^ | (0.0386) |
| *, **, *** : significant to 10%, 5% and 1%, respectively .  Standard error in parentheses. | | | | | | |
