# Supplementary material for: Arrangements with the NHS for providing healthcare services: do they improve financial performance of private for-profit hospitals in Spain?
Source: Health Econ Rev. 2021 Mar 10;11:9. doi: 10.1186/s13561-021-00304-4 (PMC7944633; doi:10.1186/s13561-021-00304-4)
Supplement: Supplementary file 3 — Additional file 3. [file 13561_2021_304_MOESM3_ESM.docx]

| **Table A3. GMM years estimation results in models M7- M12 (Contin. of Table 8)**  Dependent variable: ROA | | | | | | |
| --- | --- | --- | --- | --- | --- | --- |
|  | **M7** | **M8** | **M9** | **M10** | **M11** | **M12** |
| Sample | All hospitals | All hospitals  >=50 beds | General  All | General  >=50 beds | Medium & long stay | Medium & long stay  >=50 beds |
| Year 2001 | 0.0825^***^ | 0.0898^***^ | 0.0542^**^ | 0.0595^***^ | 0.0339 | 0.0818^**^ |
|  | (0.0205) | (0.0211) | (0.0215) | (0.0198) | -0.0237 | (0.0398) |
| Year 2002 | 0.0777^***^ | 0.0779^***^ | 0.0577^***^ | 0.0573^***^ | - | 0.0368 |
|  | (0.0194) | (0.0199) | (0.0202) | (0.0186) |  | (0.0382) |
| Year 2003 | 0.0874^***^ | 0.0894^***^ | 0.0555^***^ | 0.0626^***^ | 0.0612^***^ | 0.0816^**^ |
|  | (0.0183) | (0.0189) | (0.0190) | (0.0175) | (0.0226) | (0.0355) |
| Year 2004 | 0.0639^***^ | 0.0671^***^ | 0.0304^*^ | 0.0355^**^ | 0.0345 | 0.0601^*^ |
|  | (0.0175) | (0.0181) | (0.0181) | (0.0168) | -0.0240 | (0.0339) |
| Year 2005 | 0.0728^***^ | 0.0702^***^ | 0.0336^**^ | 0.0441^***^ | 0.0450 | 0.0516^*^ |
|  | (0.0166) | (0.0172) | (0.0170) | (0.0159) | -0.0284 | (0.0275) |
| Year 2006 | 0.0677^***^ | 0.0604^***^ | 0.0357^***^ | 0.0344^***^ | 0.0284^***^ | 0.0301^***^ |
|  | (0.0157) | (0.0166) | (0.0162) | (0.0154) | (0.0295) | (0.0266) |
| Year 2007 | 0.0684^***^ | 0.0535^***^ | 0.0336^**^ | 0.0292^**^ | 0.0154 | 0.0200 |
|  | (0.0149) | (0.0158) | (0.0153) | (0.0146) | (0.0321) | (0.0246) |
| Year 2008 | 0.0610^***^ | 0.0457^***^ | 0.0309^**^ | 0.0282^**^ | -0.0059 | - |
|  | (0.0142) | (0.0151) | (0.0146) | (0.0140) | -0.0339 |  |
| Year 2009 | 0.0440^***^ | 0.0462^***^ | 0.0343^**^ | 0.0398^***^ | -0.0314 | -0.0119 |
|  | (0.0133) | (0.0142) | (0.0137) | (0.0132) | -0.0346 | (0.0236) |
| Year 2010 | 0.0279^**^ | 0.0230^**^ | 0.0125 | 0.0227^*^ | -0.0617^*^ | -0.0600^**^ |
|  | (0.0125) | (0.0133) | (0.0129) | (0.0125) | (0.0375) | (0.0249) |
| Year 2011 | 0.0297^**^ | 0.0163 | 0.0157 | 0.0144 | -0.0617 | -0.0751^***^ |
|  | (0.0118) | (0.0126) | (0.0122) | (0.0119) | (0.0384) | (0.0259) |
| Year 2012 | 0.0266^**^ | 0.0248^**^ | 0.0077 | 0.0137 | -0.0367 | -0.0308 |
|  | (0.0114) | (0.0121) | (0.0118) | (0.0115) | (0.0411) | (0.0276) |
| Year 2013 | 0.0158 | 0.0118 | 0.0041 | 0.0205^*^ | -0.0908^**^ | -0.0908^***^ |
|  | (0.0109) | (0.0117) | (0.0113) | (0.0111) | (0.0423) | (0.0283) |
| Year 2014 | 0.0192^*^ | 0.0093 | 0.0126 | 0.0111 | -0.0688 | -0.0758^**^ |
|  | (0.0105) | (0.0112) | (0.0108) | (0.0107) | (0.0442) | (0.0301) |
| Year 2015 | 0.0117 | 0.0058 | 0.0067 | 0.0094 | -0.0910^**^ | -0.1061^***^ |
|  | (0.0101) | (0.0107) | (0.0105) | (0.0104) | (0.0462) | (0.0323) |
| Year 2016 | 0.0096 | 0.0014 | - | - | -0.0680 | -0.0877^***^ |
|  | (0.0099) | (0.0106) |  |  | (0.0475) | (0.0336) |
| Year 2017 | - | - | -0.013 | 0.0024 | -0.0700 | -0.0958 |
|  |  |  | (0.0111) | 0.0113 | (0.0485) | (0.0353) |
| *, **, *** : significant to 10%, 5% and 1%, respectively .  Standard error in parentheses.   - Eliminated because of collinearity | | | | | | |

Source: Own elaboration
